# Etomidate Versus Propofol for Monitored Anesthesia Care During Endoscopic Retrograde Cholangiopancreatography: A Prospective Randomized Controlled Trial

**DOI:** 10.7759/cureus.43178

**Published:** 2023-08-08

**Authors:** Udit Dhingra, Nitin Mantri, Soveena Pani, Deepak K Tempe, Mahesh Arora

**Affiliations:** 1 Anesthesiology, Institute of Liver and Biliary Sciences, New Delhi, IND; 2 Anesthesiology, Vishesh Jupiter Hospital, Indore, IND; 3 Anesthesiology, Govind Ballabh Pant Institute of Postgraduate Medical Education and Research, New Delhi, IND

**Keywords:** monitored anaesthesia care, intravenous sedation, haemodynamic parameters, etomidate, propofol based sedation, endoscopy ercp

## Abstract

Background and objectives

Propofol-based sedation is one of the most commonly used methods for endoscopic retrograde cholangiopancreatography (ERCP). The commonest complications during ERCP are in the form of adverse cardiopulmonary events as a result of sedation. Etomidate has a more stable cardiovascular and respiratory profile than propofol and has been used for sedation in simple gastrointestinal endoscopy but has not been studied for procedural sedation in ERCP. The objective of the present study was to compare the safety and feasibility of etomidate and propofol for sedation during ERCP procedures.

Methods

This single-center, randomized trial included 100 American Society of Anesthesiologists (ASA) physical status class I to II patients who were scheduled for ERCP. All patients received midazolam 0.02 mg/kg, lignocaine (2%) 1 mg/kg, and fentanyl 1 µg/kg intravenously, followed by etomidate or propofol according to the group allocation. The primary outcome was to compare the mean arterial pressure (MAP) at various timepoints between the two groups and secondary outcomes were to compare oxygen saturation, induction and recovery times, and adverse events. Transient hypotension was defined as any decrease in MAP below 60 mmHg or 20% below the baseline. Transient hypoxia was defined as desaturation (saturation of peripheral oxygen (SpO2) <92%) lasting for more than 10 seconds requiring airway intervention.

Results

Fifty patients were enrolled in each group (Group E: etomidate and Group P: propofol). Transient hypotension occurred in eight (16%) patients in Group P, and two (4%) patients in Group E (P= 0.045). Baseline MAP was comparable between the two groups but was significantly lower in Group P at three timepoints during the study. Nine (18 %) patients in Group P had a transient hypoxic episode, compared to none in Group E (p= 0.006). The induction and recovery times were similar in the two groups.

Conclusions

Etomidate offers better hemodynamic and respiratory stability than propofol and can be recommended for use during ERCP in ASA I/II patients.

## Introduction

Endoscopic retrograde cholangiopancreatography (ERCP) is a complex procedure for the relief of obstructive jaundice, often requiring deep sedation or general anesthesia [1]. Anesthetic goals include managing patient anxiety, discomfort, and pain, enhancing patient cooperation, and maintaining hemodynamic and respiratory stability. ERCP is mainly performed under sedation, as it provides the endoscopist with an ideal environment for the smooth completion of the procedure, minimizes the risk of physical injury during an examination, and provides faster recovery times as compared to general anesthesia [2].

An ideal sedative agent for ERCP should have a favorable pharmacokinetic profile, i.e. fast onset of action, short half-life, and good hemodynamic and respiratory stability with fast recovery. In this respect, propofol-based sedation is one of the most commonly used drugs for deep sedation in ERCP as a single agent or in combination with other medications [3]. Although propofol is routinely administered for ERCP, it has proven potential adverse events like hypoxia, hypotension, and risk of respiratory depression including apnoea [4]. An analgesic agent such as fentanyl is often added to alleviate the pain during scope manipulation and therapeutic procedures [5].

Many large multicentre studies have demonstrated that most complications during ERCP are cardiopulmonary adverse events such as hemodynamic instability, aspiration, cardiac arrest, and even death, which can be attributed to the administration of anesthetic agents outside the operating room setting [6]. So, the need for an alternative agent to propofol that provides a superior safety profile is imperative.

Etomidate has been used as a sedative for short procedures for many years [7]. It is a short-acting nonbarbiturate hypnotic agent with a rapid onset (20-50 seconds) and offset of action (5-15 minutes) with a more stable cardiovascular and respiratory profile than propofol [8]. It does not inhibit the sympathetic tone, thereby avoiding drastic changes in blood pressure, heart rate (HR), and respiratory rate [9]. Likewise, apnea and histamine release are significantly lesser with etomidate compared to propofol [10]. Etomidate may cause side effects such as pain on injection, postoperative nausea and vomiting (PONV), dose-dependent myoclonus, and adrenocortical suppression which is temporary and resolves within 24 hours of induction. Considering the pharmacological characteristics of etomidate, it may prove to be a promising agent as an alternative to propofol in patients undergoing advanced endoscopic procedures such as ERCP [10].

In our institute, anesthesiologist-driven sedation is the mandate for all patients undergoing ERCP. To test the hemodynamic and respiratory responses of etomidate as an induction and maintenance agent, American Society of Anesthesiologist (ASA) physical status class I/II patients undergoing ERCP were included in the present study.

## Materials and methods

This prospective randomized controlled study was conducted in a tertiary care hospital from August 2022 to October 2022 after obtaining approval from the Institutional Ethics Committee of the Institute of Liver and Biliary Science, New Delhi, India (approval number: IEC/2022/92/MA07), the trial was registered in the Clinical Trials Registry of India (CTRI/2022/07/043892). Written informed consent was taken from all the patients, following the principles of the Declaration of Helsinki. A total of 100 patients (aged 18-70 years) of either sex, ASA grade I -II, undergoing ERCP were randomized into two groups of 50 patients each using computer-generated randomization: (i) Group E, which received etomidate as a sedative agent, and (ii) Group P, which received propofol. Allocation concealment was done via a sequentially numbered sealed envelope technique. Patient refusal, known allergy to the study drugs, uncontrolled diabetes, known cases of cirrhosis, hemodynamic instability, post-liver transplant recipients, porphyria, pre-existing epilepsy, pregnancy, lactation, and patients with known adrenocortical insufficiency were excluded. The primary outcome of the study was to compare mean arterial pressure (MAP) at various time points between the two groups. The secondary outcomes were to compare respiratory parameters, induction, recovery time, and adverse events between the two groups.

A routine pre-operative evaluation was performed on the patient's arrival at the endoscopy complex. In the endoscopy procedure room, routine ASA monitoring including electrocardiogram (ECG), non-invasive blood pressure (NIBP), pulse oximetry (saturation of peripheral oxygen (SpO2)), and Bispectral Index (BIS) were attached to the patient, and baseline recordings were taken. Patients were premedicated with midazolam 0.02 mg/kg, lignocaine (2%) 1 mg/kg, and fentanyl 1 µg/kg, five minutes before the procedure. Patients were given the prone position with all precautions and oxygenation was started through a nasal cannula 5litre/minute. Group E received etomidate 0.15-0.2 mg/kg over 60 seconds to achieve a BIS value between 50 and 60 followed by an infusion at 5 -10 µg/kg/min via an infusion pump. Group P received propofol 1.5-2 mg/kg over 60 seconds to achieve a BIS value between 50 and 60 followed by an infusion at 50-75 µg/kg/min via an infusion pump. After induction, the operator was allowed to insert the endoscope, and sedation was titrated to a BIS value between 50 and 60. Hemodynamic and respiratory parameters including heart rate, mean arterial pressure (MAP), SpO2, and respiratory rate were recorded at baseline (on arrival in the endoscopy room (T=0), five minutes after premedication (T=1) when BIS was 50-60 (T=2), and thereafter every five-minute interval (T3-T12) during ERCP. The anesthesia was maintained by adjusting the infusion rate of the respective anesthetic agent to achieve the target BIS value of 50-60. Any patient movement was treated with a bolus of the induction agent, propofol 0.2 mg/kg or etomidate 0.04mg/kg in the respective groups. Any decrease in the MAP below 60 mmHg or 20% below the baseline was treated with IV ephedrine 3-6 mg bolus, 0.6 mg atropine was administered when the heart rate decreased below 50 beats per minute, and for persistent tachycardia above 100 beats per minute lasting for five minute, fentanyl bolus (0.5 µg/kg) was administered. If spontaneous ventilation was insufficient to maintain the SpO2 greater than 92%, the anesthesiologist gave a jaw thrust maneuver to assist, and on further desaturation, mask ventilation was instituted.

Statistical analysis

The sample size was calculated based on the result of a similar study by Song et al. [11], in which the average percent change to baseline in MAP was -7.5±8.3 (n = 40) in the etomidate group and -13.5±10.3 (n = 40) in the propofol group. With a significance level of 5% and power of 80%, the sample size was calculated to be 47 for each group. Adding a safety factor to account for drop-offs, 50 participants for each group were considered for this study. The continuous data are presented as mean ± SD. Categorical data are presented as percentages. Continuous data were analyzed using an independent T-test. Categorical data were analyzed using the Chi-square test. A p-value < 0.05 was considered significant. Changes in the vital signs against elapsed sedation time were analyzed using scatter plots and locally weighted polynomial regression. The statistical analysis was performed using IBM SPSS Statistics for Windows, Version 26.0 (Released 2019; IBM Corp., Armonk, New York, United States).

Definitions

A major adverse event was a need for endotracheal intubation, permanent neurologic impairment, or death. The transient hypoxic event was defined as a decrease in SpO2 <92% lasting for more than 10 seconds, which required airway intervention such as chin-lift/jaw thrust maneuver, an increase in oxygen flow, or assisted ventilation. Adverse cardiovascular events included tachycardia (heart rate > 100 beats/min), bradycardia (heart rate < 50 beats/min), hypotension, and arrhythmia. Transient hypotension was defined as MAP < 60 mm Hg or a more than 20% decrease from the baseline requiring intervention in the form of IV ephedrine 3-6 mg. Arrhythmia was defined as any rhythm that was not normal sinus rhythm with normal atrioventricular conduction. Percent change in MAP from the baseline was calculated at various time points as the difference between baseline MAP (T0) subtracted by the MAP at various time points (T1-12) divided by the baseline MAP.

Induction time was defined as the time interval between the start of etomidate or propofol administration to when a BIS value of 50-60 was recorded. Total procedure time was defined as the time from endoscope insertion to endoscope withdrawal. Recovery time was defined as the time interval between the endoscope removal and the patient's full recovery (Aldrete score of 10) [12]. Myoclonic movements were defined as involuntary short muscle contractions leading to short observable movements in parts of the body.

## Results

**Figure 1:**
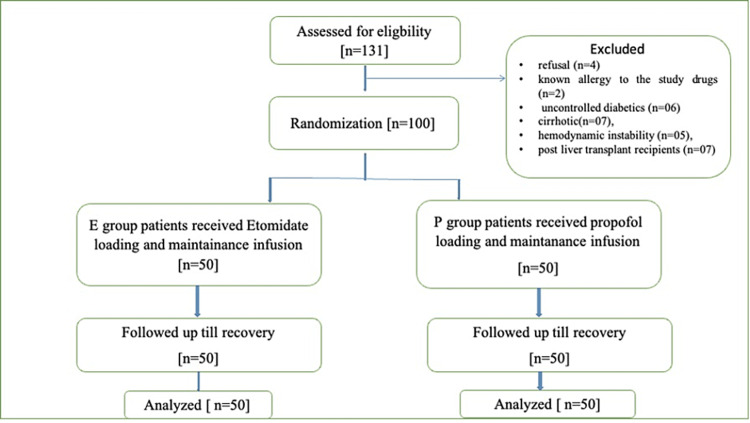
CONSORT diagram of the randomized trial. CONSORT: Consolidated Standards of Reporting Trials

The flow of patients through the trial is depicted in Figure 1. In the present study, 131 patients were enrolled; among these, 31 patients were excluded for various reasons including refusal (n=4), known allergy to the study drugs (n=2), uncontrolled diabetics (n=6), cirrhosis (n=7), hemodynamic instability (n=5), and post-liver transplant recipients (n=7). Hence, a total of 100 patients were eligible for analysis. Random sequencing was performed and 50 patients each were allocated to groups P and E. There was no loss to follow-up during the study.

Baseline characteristics

**Figure 2:**
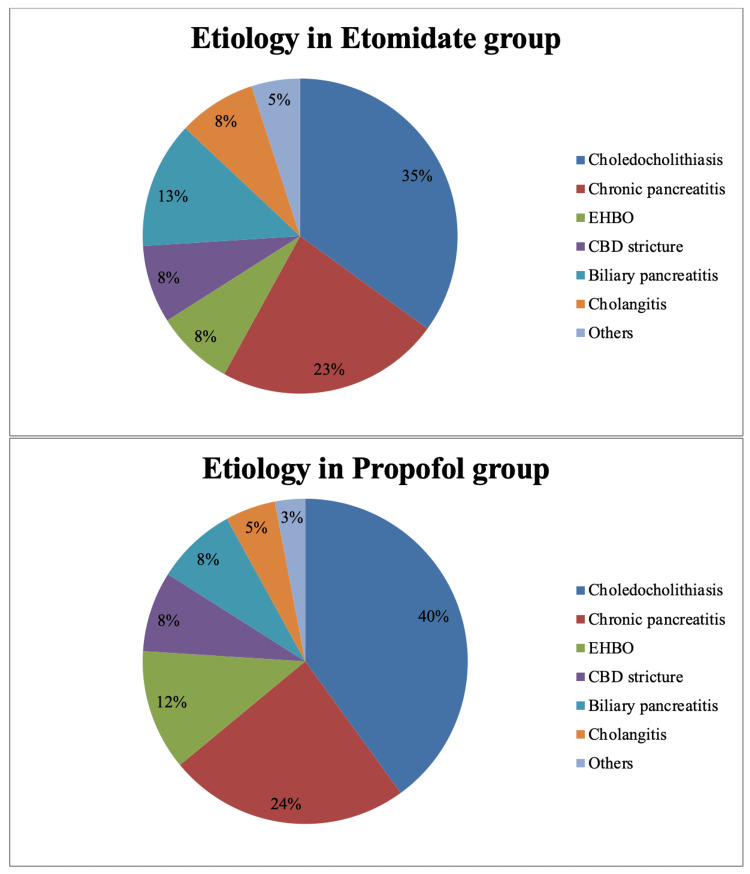
Etiology of patients undergoing ERCP EHBO: extrahepatic biliary obstruction; CBD: common bile duct; ERCP: endoscopic retrograde cholangiopancreatography

Demographic data are shown in Table 1. The mean age was 48.9 ± 13.01 years and 48.8 ± 14.3 years in the propofol and etomidate groups, respectively (P=0.965). There was no significant difference in gender, body mass index, presence of comorbidity, ASA grading, modified Malampatti (MMP) grades, and baseline hemodynamic parameters (heart rate, MAP, SpO2, respiratory rate). The etiology of the patients undergoing ERCP has been presented in Figure 2.

**Table 1:** Demography of the study population Data are expressed as mean±SD or the number of patients. BMI: body mass index; ASA: American Society of Anesthesiologists; MMP: modified Mallampatti score; BPM: beats per minute; RPM: rate per minute; SPO2: saturation of peripheral oxygen

Variables	Group P (n= 50)	Group E (n=50)	p-value
Age (in years)	48.9 ± 13.01	48.8 ± 14.3	0.965
Female	16 (32%)	16 (32%)	1.00
Male	34 (68%)	34 (68%)	1.00
BMI (kg/m^2^)	24.39±3.86	24.0±4.35	0.640
Comorbidity	11 (22%)	15 (30%)	0.490
ASA Grade I	37 (74%)	35 (70%)	0.824
ASA Grade II	13 (26%)	15 (30%)	0.82
MMP class II	48 (96%)	47 (94%)	0.617
MMP class III	2 (4%)	3 (6%)	0.82
Heart rate (bpm)	83.16±12.31	81.50±11.34	0.485
Mean arterial pressure (mm of Hg)	85.42±8.50	83.84±8.38	0.352
SpO_2_ (%)	99.20±1.19	99.30±1.23	0.681
Respiratory rate (rpm)	17.44±2.67	17.26±3.56	0.681

Procedure characteristics

The procedure and outcomes are presented in Table 2. The procedure duration between both groups was comparable (35.4 ±11.1 minutes and 32.1±12.1 minutes for propofol and etomidate, respectively). The induction and recovery times were comparable between the two groups; induction time was 131.5±14.1 seconds and 133.3±15.1 seconds for propofol and etomidate, respectively (P =0.537), and recovery time was 6.8±1.8 minutes and 6.5±1.8 minutes for propofol and etomidate, respectively (P= 0.454). The mean propofol dosage requirement was 8.32±2.65 mg/kg/hour, whereas the mean etomidate dose requirement was 1.08±0.32 mg/kg/hour.

**Table 2:** Procedure and outcomes Data are expressed as Mean ± SD

Variable	Group P (n=50)	Group E (n=50)	p-value
Induction time (second)	131.520±14.164	133.340±15.197	0.537
Procedure duration (minutes)	35.469±11.132	32.180±12.107	0.163
Recovery time (minutes)	6.857±1.814	6.580±1.852	0.454
Mean dose (mg/kg/hour)	8.32±2.65	1.08±0.32	

Hemodynamic variations

**Figure 3:**
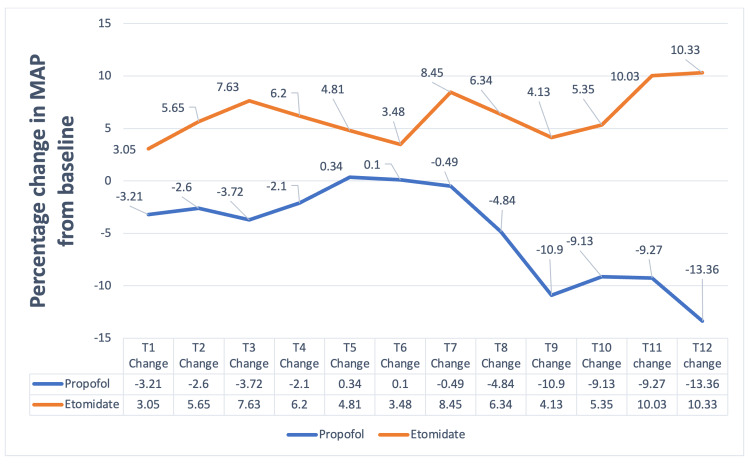
Change in mean arterial pressure T1 = At five minutes after patients received premedication; T2= BIS recorded was 50-60 after induction, T3-12= five-minute intervals during endoscopic retrograde cholangiopancreatography (ERCP)

The heart rate, SpO2, and respiratory rate were comparable at various timepoints in both groups. The MAP was significantly lower in Group P at three timepoints: T2 (p=0.028), T3 (p=0.001), and T11 (p=0.018) (Table 3) compared to Group E. Group P had a significant percentage fall in MAP from baseline as compared to Group E at various timepoints (p-value = 0.001) (Figure 3).

**Table 3:** Mean arterial pressure over designated time points Data are expressed as mean ± SD

Time (minutes)	Group P (n=50), mm Hg	Group E (n=50), mm Hg	p-value
T0	85.42±8.50	83.84±8.38	0.352
T1	82.68±11.96	86.40±10.48	0.109
T2	83.20±10.48	88.58±13.23	0.028
T3	82.24±11.17	90.24±12.90	0.001
T4	83.56±14.07	89.04±14.07	0.053
T5	85.71±14.27	87.87±14.28	0.0517
T6	85.51±13.87	86.76±10.98	0.708
T7	85.00±11.66	90.93±9.39	0.108
T8	81.28±9.44	89.16±11.28	0.064
T9	76.10±11.93	87.30±14.12	0.072
T10	77.62±7.55	88.33±11.60	0.058
T11	77.50±6.60	92.25±6.23	0.018
T12	74.00±6.24	92.50±7.77	0.058

Adverse events

There was no major adverse cardiovascular or respiratory event. Eight patients (16%) in Group P had a transient hypotensive episode that required intervention, compared to two (4%) patients in Group E (P= 0.045). Nine (18 %) patients in Group P had a transient hypoxic episode managed by jaw thrust (10%) or mask ventilation (8%), compared to none in Group E (P= 0.006). The incidence of myoclonus was distinctively higher in Group E as compared to Group P (0 vs 12%; P <0.001). Propofol caused pain on injection leading to discomfort to patients (propofol vs etomidate: 10% vs 0%; P <0.056) (Table 4). Other adverse events reported were of no significance and required no active intervention.

**Table 4:** Adverse events All data is expressed in the number of patients (percentage).

Variables	Group P (n=50)	Group E (n=50)	p-value
Bradycardia	2 (4%)	0	0.495
Tachycardia	5 (10%)	2 (4%)	0.95
Patient movement	24 (48%)	17 (34%)	0.222
Transient hypotensive episodes	8 (16%)	2 (4%)	0.045
Transient hypoxic episodes	9 (18%)	0	0.006
Myoclonus	0	6 (12%)	0.001
Nausea	0	1 (2%)	1
Pain on injection	5 (10%)	0	0.056

## Discussion

In this randomized study comparing etomidate with propofol as sedatives for ERCP procedures, we showed patients in the propofol group had a higher incidence of transient hypotensive episodes and hypoxic episodes that required treatment and the decrease in blood pressure compared to baseline was significantly higher in the propofol group than in the etomidate group. Both the groups had similar induction and recovery times; in addition, propofol caused a higher incidence of pain on injection. Thus, the use of etomidate for procedural sedation in ASA class I/II for ERCP offered the benefit of additional hemodynamic and respiratory stability.

Previous studies that used etomidate for diagnostic purposes of shorter duration like colonoscopy and upper gastrointestinal endoscopy have demonstrated that etomidate offers stable hemodynamic and respiratory conditions [7], but its usage in ERCP is still uncertain as its use as an infusion for prolonged procedures has not been described frequently.

Etomidate has a more stable cardiovascular profile in comparison with propofol as it does not inhibit the contractile function of the myocardium [10]. This was seen in the present study where etomidate anesthesia during ERCP caused more stable hemodynamic responses compared with propofol. Similar beneficial results have been reported in earlier studies by Song et al. and Park et al. in patients undergoing ERCP [11,13]. In the study by Park et al., tachycardia was reported as a significant event in the etomidate group, in our study there was no clinical difference between the heart rates noted in both groups. However, tachycardia was noted in both groups but did not require any intervention. Such events may be contributed by procedure factors such as scope insertion and manipulation.

Hypoxia is the most serious side effect of propofol sedation [1], with previous studies with propofol showing that even though administered by an experienced anesthesiologist, 42.8% of patients sedated for ERCP with propofol and fentanyl experienced transient hypoxia [14]. Akhondzadeh et al. reported a much higher incidence of patients (63%) experiencing apnoea in patients with propofol-fentanyl sedation [15]. By virtue of its limited suppression of ventilation, etomidate offers a distinct advantage over propofol. This was seen in the present study where none of the patients in the etomidate group had any episode of desaturation. Similar beneficial results have been reported in earlier studies by Park et al. and Kim et al. in patients undergoing advanced endoscopic procedures [13,16]. These studies used a bolus dose of the induction agents followed by bolus as per need while observing for patient awakening. In our study, a bolus dose of induction agent was followed by infusion titrated to BIS, and an additional bolus was used only on patient movement. 

Etomidate has a propensity to cause myoclonic movements. Etomidate acts at the gamma-aminobutyric acid (GABA)-A receptor, pathways that control skeletal muscles, and spontaneous neuronal discharge are sensitized by cutting GABA neuronal transmission and cause myoclonic contraction [17]. The incidence of myoclonus has been shown to increase with the speed of etomidate administration and dose duration [18]. The myoclonus can be ameliorated by pre-treatment with lignocaine and midazolam and slower administration of the bolus dose [19,20]. In the present study, the incidence of myoclonus is 12%, which is much less than the earlier reported incidence of 40-50% in unpremedicated patients [19,20].

Pain on injection is a frequent complaint, occurring in up to 30% of patients receiving an intravenous bolus of propofol [21]. In our study, propofol caused a significantly higher incidence of pain than etomidate. Similar results were shown in a study by Song et al. in patients undergoing ERCP [11]. Only one patient in the etomidate group had nausea post-procedure. After one hour of observation, symptoms improved and no intervention was required. It has been previously reported that a single dose of etomidate can lead to a decrease in adrenal function; however, this effect appears to be transient and has not been associated with long-term adrenal suppression or increased mortality, according to several previous studies [22,23].

As the applications of ERCP are expanding, elderly patients who are susceptible to cardiovascular and respiratory instability either due to existing comorbidities, advanced age, sepsis due to cholangitis, and underlying etiology such as malignancy are undergoing therapeutic ERCP [24]. These interventions are performed outside the operating room where serious complications are not unknown. Hence, it is important to use anesthetic agents that provide safety to patients.

The present paper demonstrates that etomidate can be one such agent. The major undesirable side effect of etomidate, myoclonus can be reduced by pre-treatment with lignocaine and midazolam as shown by previous studies [19,20]. In our study, etomidate was used as an infusion by titrating its dose according to BIS values. Routinely BIS is not available in the endoscopy suite, in its absence a bolus dose of etomidate of 0.15-0.2 mg/kg over 60 seconds can be followed by an infusion at 7 -10 µg/kg/minute via an infusion pump, which can be titrated to patient awakening based on modified observer's assessment of alertness and sedation (MOAA/S) scale [25].

There were certain limitations to this study. First, the anaesthesiologist could not be blinded to group allocation because this was a study comparing etomidate and propofol, which have to be administered at a different infusion rate. Second, the trial was a single-center study, and further multi-centric, prospective research may be necessary. Third, the cohort of patients included in the study are ASA I and II, and outcomes of class ASA III/IV and elderly patients could not be studied and further studies need to be conducted on these cohorts of patients.

## Conclusions

The use of etomidate as a sedative agent for ERCP provided hemodynamically superior conditions than the use of propofol with patients experiencing lesser transient hypotensive episodes. Etomidate use also provided respiratory stability with no patient having transient hypoxic episodes during the procedure. There was no difference in the patient recovery and induction times in both groups. Thus, etomidate can be used for sedation in ASA I/II patients undergoing ERCP under monitored anesthesia care with the benefits of hemodynamic and respiratory stability with similar recovery profiles as compared to propofol.
